# Short-term suborbital space flight curtails astronauts’ dopamine levels increasing cortisol/BDNF and prompting pro-oxidative/inflammatory milieu

**DOI:** 10.1186/s40779-025-00589-0

**Published:** 2025-01-20

**Authors:** Gerardo Bosco, Angelo Landolfi, Tommaso Antonio Giacon, Alessandra Vezzoli, Nazareno Paolocci, Simona Mrakic-Sposta

**Affiliations:** 1 Department of Biomedical Sciences, University of Padova, 35129 Padua, Italy; 2 Italian Air Force Medical Service, 00175 Rome, Italy; 3 Institute of Clinical Physiology, National Research Council (IFC-CNR), 20162 Milan, Italy; 4 Division of Cardiology, Johns Hopkins Medical Institutions, Baltimore, MD 21224 USA

**Keywords:** Suborbital space flight, Oxidative stress and inflammation, Neuroplasticity

Dear Editor,

Space flight (SF) is substantially increasing at present. The emergence of commercial suborbital SF, such as the Virgin Galactic with VSS Unity and VMS Eve spacecraft, is extending to civilians, being previously confined to military and/or professional astronauts only. This new evidence offers additional opportunities for better characterizing the impact that the transition from Earth’s 1G to microgravity in space could have on the astronauts’ health while comparing well-trained subjects such as the latter to space newcomers [1].

In the current pilot study, we developed an assay that is easy to perform based on the collection of saliva samples during SF. “Galactic 01” was carried out with the VSS Unity spacecraft (SpaceShipTwo class), which was launched from Virgin Galactic’s Spaceport-America in New Mexico (USA) at 8:30 local time. The flight began after a 60-min ascent, reaching a speed of Mach 2.88 and a peak altitude of 282,000 feet, with accelerations of + 4G along the Z and X axes. During the microgravity phase (3:09 min), the spacecraft rotated 180° and the crew experienced weightlessness. The descent included re-entry (with maximum accelerations of + 4Gz and + 1Gx) and gliding, concluding with a landing at Spaceport America after 72 min (Fig. 1a).

**Fig. 1:**
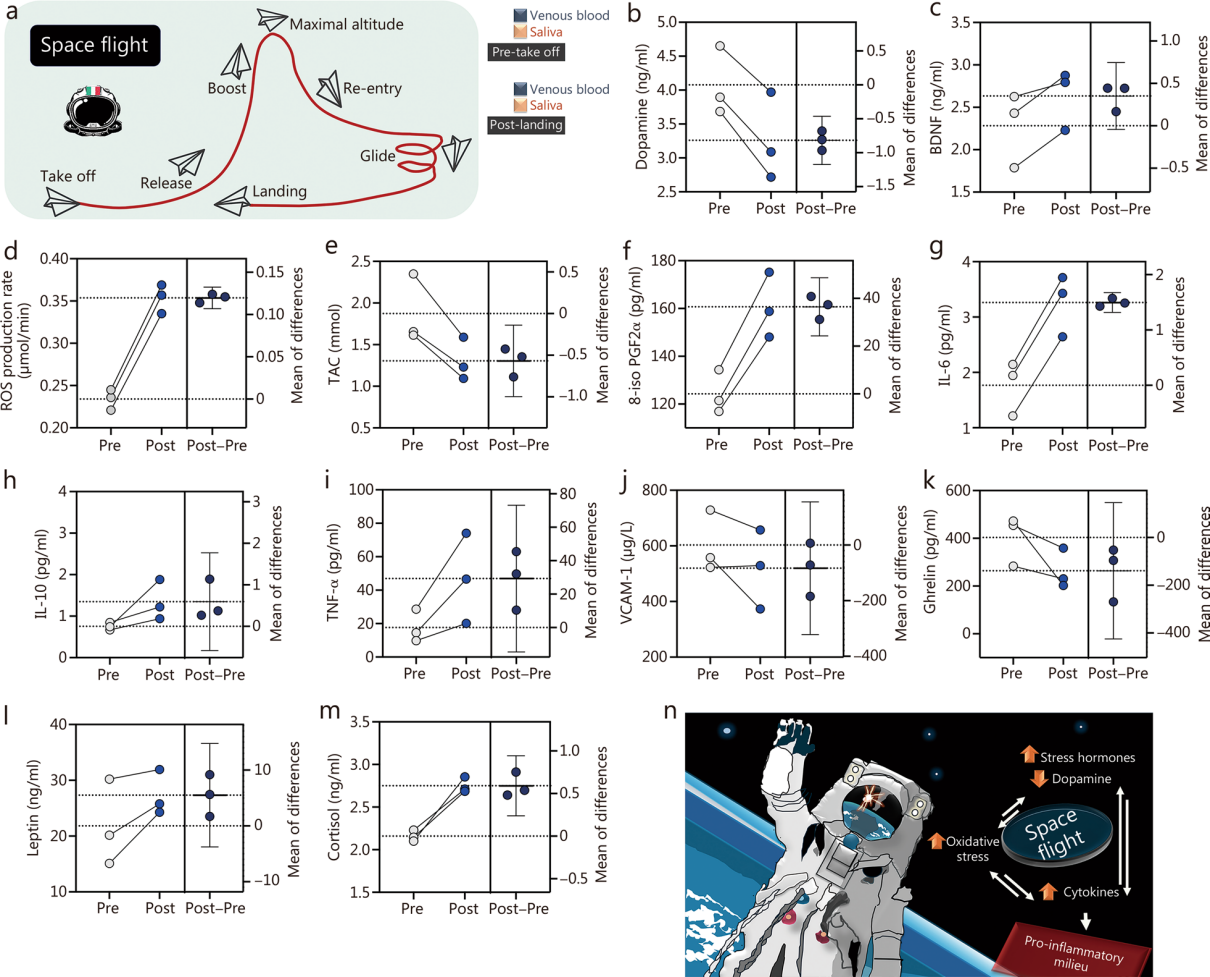
Characteristics of short-term suborbital space flight (SF) in astronauts. **a** SF steps: from taking off to landing, modified from https://www.virgingalactic.com. Individual lines, mean plots, and (mean ± SD) at pre- and post-suborbital SF are shown. **b, c** Neuroprotection: dopamine and brain-derived neurotrophic factor (BDNF). **d–j** Biomarkers of oxidative stress, inflammation concentration, and endothelial dysfunction: reactive oxygen species (ROS), total antioxidant capacity (TAC), 8-iso prostaglandin F2α (8-iso PGF2α), interleukin (IL)−6, IL-10, tumor necrosis factor-α (TNF-α), and vascular cell adhesion protein-1 (VCAM-1). **k–m** Hormone secretions: ghrelin, leptin, and cortisol. **n** Synopsis of the study’s findings on three astronauts after a short-term (approximately 72 min) SF and overall conceptual framework of the study. Figures created with GraphPad Prism (GraphPad Software Inc. California, USA, version 10.3.0, https://www.graphpad.com/)

Three mission specialists [age: (47.0 ± 3.36) years; height: (179.0 ± 10.44) cm, and weight: (78.67 ± 11.37) kg] in good health conditions, who were medically certified fit to fly according to European Union Aviation Safety Agency (EASA) Class 2 regulation, were studied before and immediately after navigation. In doing so, we chose to delimit the problem presented by the wide spectra of possible SF-imparted behavioral and structural alterations to 4 main investigative fields: 1) the status of factors involved in cognition and body response to stress [dopamine, brain-derived neurotrophic factor (BDNF), and cortisol]; 2) multiple markers of oxidative stress (from different body parts) to eventually portray a generalized pro-oxidative body condition; 3) pro-inflammatory metrics [i.e., systemic levels of interleukin (IL)−6, IL-10, tumor necrosis factor-α (TNF-α)], as well early markers of endothelial dysfunction, such as vascular cell adhesion protein-1 (VCAM-1); 4) indexes of hormone imbalance controlling not only appetite/nutrition but also oxidative stress and having potential long-term predictive value when eventual cerebral and cardiovascular accidents are concerned (i.e., ghrelin and leptin levels) (Additional file 1: Materials and methods).

As depicted in Fig. 1b, dopamine levels decreased by 19%, while BDNF levels increased by 15% (Fig. 1c) in astronauts’ saliva after SF. Dopamine and BDNF are interrelated, with the activation of dopamine receptors promoting BDNF expression, while BDNF supports the survival of dopaminergic neurons. The increase in BDNF may offer neuroprotective benefits against conditions such as ischemia and neurotoxicity. These findings highlight the differential effects of SF on dopamine and BDNF in an accessible extracellular fluid [2]. Upon returning to Earth, astronauts exhibit oxidative stress and a pro-inflammatory state, with elevated reactive oxygen species (ROS) levels (+ 52%), assessed by electron paramagnetic resonance (EPR) techniques (Fig. 1d), and reduced total antioxidant capacity (TAC, –32%), indicating a redox imbalance (Fig. 1e). Oxidative stress, which is particularly harmful to neuronal cells, is well-documented in microgravity-exposed rodents but fewer so in astronauts [3]. Lipid peroxidation marker 8-iso prostaglandin F2α (8-iso PGF2α) was also observed post-SF (Fig. 1f), thus providing the first evidence of a systemic redox imbalance in astronauts after SF. Post-SF, astronauts also showed an increase in pro-inflammatory markers, with IL-6 (+ 85%) (Fig. 1g), IL-10 (+ 79%) (Fig. 1h), and TNF-α (+ 161%) (Fig. 1i). Conversely, VCAM-1, an indicator of endothelial dysfunction related to cardiovascular disorders, slightly decreased (–14%) (Fig. 1j), suggesting an emerging pro-inflammatory state upon return to Earth, although there is no clear evidence of endothelial dysfunction. These findings underscore potential inflammation-related risks for astronauts after SF [4]. Additionally, SF alters the hormones associated with appetite and stress, likely contributing to oxidative stress [5]. Ghrelin, an antioxidant hormone, declined by 35% (Fig. 1k), while leptin, a pro-inflammatory and pro-oxidative agent, increased by 25% (Fig. 1l). Cortisol, a stress hormone known for amplifying oxidative stress, rose significantly (+ 51%) (Fig. 1m). Therefore, a hormonal imbalance after SF may exacerbate oxidative stress in astronauts.

This pilot study revealed that SF induces a pro-oxidative/inflammatory environment in astronauts **(**Fig. 1n**)**, along with reduced dopamine and elevated stress hormones. All these factors can eventually converge to cause systemic stress and impaired cognitive function after repeated/longer exposures [3, 4]. Admittedly, we did not conduct cognitive/behavioral tests. However, a recent study demonstrated that a 3-day spaceflight is not potent enough to affect cognitive performance in civilians [5]. Other remaining questions to consider are whether SF has an impact on the neurohormonal assets of female and crew members of different ages. Moreover, for a more detailed understanding of the involved pathways, studies could be conducted using consolidated animal models of microgravity, such as exploring a broader spectrum of hormones and pro-oxidative/inflammatory factors. In short, the current glimpse obtained within a 72-min time window showing incipient oxidative/inflammatory stress would help guide future more time-extended studies to determine what the central and peripheral consequences of these initial alterations could be. 

## Supplementary Information

**Additional file 1.** Materials and methods. **Fig. S1** Stacked plots of the reactive oxygen species (ROS) by electron paramagnetic resonance (EPR) spectra recorded at 37 °C, the signal comes from the reaction of 1-hydroxy-3-carboxymethyl-2,2,5,5-tetramethyl-pyrrolidine spin probe (CMH, EPR silent) to 3-carboxymethyl-2,2,5,5-tetramethyl-pyrrolidinyloxy radical (CM, EPR active).

## Data Availability

The datasets used and analyzed during the current study are available from the corresponding author upon reasonable request.

## Abbreviations

BDNF: Brain-derived neurotrophic factor
IL: Interleukin
8-iso PGF2α: 8-iso prostaglandin F2α
ROS: Reactive oxygen species
SF: Space flight
TAC: Total antioxidant capacity
TNF-α: Tumor necrosis factor-α
VCAM-1: Vascular cell adhesion protein-1
