# Supplementary material for: Short-term suborbital space flight curtails astronauts’ dopamine levels increasing cortisol/BDNF and prompting pro-oxidative/inflammatory milieu
Source: Mil Med Res. 2025 Jan 20;12:2. doi: 10.1186/s40779-025-00589-0 (PMC11744899; doi:10.1186/s40779-025-00589-0)
Supplement: Supplementary file 1 — Additional file 1. Materials and methods. Fig. S1 Stacked plots of the reactive oxygen species (ROS) by electron paramagnetic resonance (EPR) spectra recorded at 37 °C, the signal comes from the reaction of 1-hydroxy-3-carboxymethyl-2,2,5,5-tetramethyl-pyrrolidine spin probe (CMH, EPR silent) to 3-carboxymethyl-2,2,5,5-tetramethyl-pyrrolidinyloxy radical (CM, EPR active). [file 40779_2025_589_MOESM1_ESM.pdf]

Materials and methods

Human subjects enrolled in the study

The crew of suborbital space flight (SF) “Galactic 01” consisted of three expert male mission specialists, one Virgin Galactic crew member, and two space pilots: 1st 43 years old; 2nd 49 years old; 3rd 49 years old; height and weight: 1st 184 cm × 88 kg; 2nd 167 cm × 66 kg; 3rd 186 cm × 82 kg. The crew had numerous hours of flying experience and had undergone hypobaric and centrifuge training. They were selected among other candidates to participate in the first entire Italian crew’s suborbital flight [1].

Suborbital SF conditions

The suborbital SF “Galactic 01” was conducted using the VSS Unity spacecraft (SpaceShipTwo class), which was launched from Virgin Galactic’s Spaceport-America in New Mexico (USA) at 8:30 local time and lasted approximately about 72 min. The take-off took place with the carrier aircraft “Eve” (of the WhiteKnightTwo class). After approximately 60 min of flight, “Unity” was dropped at an altitude of roughly 44,500 feet. The suborbital shuttle operated by Virgin Galactic pilots, activated its thruster and aimed upwards, exceeding a speed of Mach 2.88 and reaching a maximum altitude of approximately 282,000 feet. The parabolic flight performed by “Unity” can be divided, for easier understanding of the scientific activity carried out, into three main phases: 1) take-off and ascent phase; 2) microgravity phase; and 3) gliding and landing phase. The take-off and climb phase can be further divided into two parts: the first part commences with the aircraft in the “Eve” - “Unity”. During this phase, which began at 8:30 local time and lasted approximately 60 min, the crew experienced a brief acceleration along the Z axis of approximately +1G. In contrast, no accelerations were detected along the X and Y axes. The second part began at 9:28 with the release of the “Unity” spacecraft and the subsequent activation of the rocket booster by the latter. At this moment, the propulsion phase began, taking the spacecraft to an altitude of 282,000 feet, representing the flight’s apogee. During this phase (approximately 1:10 min), the spacecraft reached a speed of 2.88 Mach, and the crew was subjected to

accelerations of +4G along the Z and X axes. The second phase was initiated with the exhaustion of the rocket's propulsive thrust and reaching an altitude of 282,000 feet. At the apogee, the spacecraft performed a 180° rotation on itself, starting the microgravity phase. During this phase (3:09 min), the crew experienced floating in microgravity. The 180° rotation placed the spacecraft in a descent attitude, with the nose facing the Earth, initiating the third phase of the flight. The latter can be subdivided into two phases: the re-entry phase and the gliding phase. During the re-entry phase, as the spacecraft descended to an altitude of 50,000 feet, the crew was subjected to accelerations of +4Gz and +1Gx. With the gliding phase, the flight concluded with the spacecraft landing at Spaceport America at 9:42. Even during the glide, the crew experienced accelerative forces, namely +2Gz and +1Gx. The mission lasted a total of approximately 72:00 min.

Blood and saliva collection

Pre-SF, blood and saliva samples were collected at the same time (at 5:30 am) for all three mission specialists just before the 72 min SF within the mission specialist dressing rooms of the VG Spaceport America upon completing the preparation for the wearable monitoring experiments. After SF and the landing of SpaceShip Two, blood samples were collected from the three mission specialists inside the cabin of the SpaceShip Two while it was still on the spaceport runway.

Blood venous samples were extracted from the antecubital vein using heparinized vacuum tubes (Vacutainer, Becton Dickinson, USA). The collected blood was centrifuged for 5 min at 3000× *g* to separate the plasma.

For saliva sampling, subjects were instructed regarding the correct utilization of a Salivette device (Sarstedt, Nümbrecht, Germany) and were advised to refrain from drinking, eating, smoking, brushing their teeth, and using a mouthwash during the 30-minute before salivary collection. Approximately 1 ml of saliva was collected. Before storage, saliva samples were centrifuged at 3000 rpm for 20 min. After centrifugation, both saliva and blood samples were aliquoted in cryovials and stored: plasma at –80 °C and saliva at –20 °C. Samples were transferred from the USA to Italy by a courier specialized in maintaining the cold chain, thus respecting the safety criteria. Upon arrival in the Italian laboratory

(IFC-CNR), samples were immediately analyzed for the selected biomarkers. Specifically, from saliva samples: dopamine, reactive oxygen species (ROS), total antioxidant capacity (TAC), cortisol, ghrelin, and leptin; from plasma samples: brain-derived neurotrophic factor (BDNF), ROS, 8-iso-prostaglandin F2α (8-iso-PGF2α), interleukin (IL)-6, IL-10, tumor necrosis factor-α (TNF-α), vascular cell adhesion protein-1 (VCAM-1).

Biomarkers assessment

BDNF and dopamine

BDNF was detected in plasma using an enzyme-linked immunosorbent assay (ELISA) method according to the manufacturer's instructions (Human BDNF ELISA kit, Abcam, USA), as previously described [2,3]. Tertiary antibodies were conjugated to horseradish peroxidase. Wells were developed with tetramethylbenzidine and measured at 450 nm. The BDNF content was quantified against a standard curve calibrated with known amounts of protein. Dopamine in saliva was determined by a kit (Cat. No. EU0392, Fine Test, Wuhan, China) based on a competitive ELISA detection method. The analysis was carried out in accordance with the manufacturer's instructions, as previously described [4]. The dopamine concentration was determined employing a standard curve. Samples and standards were spectrophotometrically read at a wavelength of 450 nm.

Oxidative stress and antioxidant levels

ROS measurements by electron paramagnetic resonance (EPR) spectroscopy. An X-band EPR spectroscopy (9.3 GHz; E-Scan, Bruker Co., Billerica, MA, USA) was utilized to detect ROS production in plasma and saliva samples at 37 °C using a Temperature Controller unit (Noxigen Science Transfer & Diagnostics GmbH, Elzach, Germany), interfaced with the spectrometer. The methods were previously described [5]. Spin probe 1-hydroxy-3-methoxy-carbonyl-2,2,5,5-tetramethylpyrrolidine (CMH) was employed, and a stable radical 3-carboxy2,2,5,5-tetramethyl-1-pyrrolidi-nyloxy (CP) was adopted as an external reference to convert ROS determinations into absolute quantitative values (µmol/min) **(Additional file 1: Fig. S1)**.

8-iso-PGF2α levels. Lipid peroxidation was assessed in plasma by competitive immunoassay of 8-iso-PGF2α concentration (No. 516360, Cayman Chemical, Ann Arbor, MI, USA). The concentrations of 8-iso-PGF2α were determined using a standard curve. Samples and standards were read at a wavelength of 412 nm. This method has been previously described [5]

TAC. Antioxidant capacity assay, a widely used kit-based commercial method (No. 709001, Cayman Chemical, Ann Arbor, MI, USA) was performed as previously described 6]. Briefly, 10 µl of saliva was added in duplicate to 10 µl of metmyoglobin and 150 µl of the chromogen solution. Then, reactions were initiated by the addition of 40 µl of H_2O_2, as indicated in the instructions. Reaction mixtures were incubated at room temperature for 3 min. Samples were measured at a wavelength of 750 nm.

Inflammatory and vascular endothelial status

The levels of IL-6, IL-10, and TNF-α in plasma were determined by ELISA kits (IL-6: No. 501030, Cayman Chemical, Ann Arbor, MI, USA; IL-10: Cat. No. EH0173, Fine Test, Wuhan, China; TNF-α: Cat. No. EH0302, Fine Test, Wuhan, China), according to the manufacturer's instructions. These methods were previously described [6,7]. The concentration of VCAM-1 in plasma was obtained using an ELISA kit (Cat. No. EH0326, Fine Test, Wuhan, China) in accordance with the manufacturer's instructions. Samples were measured at 450 nm. The concentration of VCAM-1 was calculated by drawing a standard curve.

Hormones

The free cortisol concentration in saliva was quantitatively determined via an ELISA method according to the manufacturer's protocol ELISA Kit (No. 500360, Cayman Chemical, Ann Arbor, MI, USA) as previously described [8]. The levels of leptin and ghrelin in saliva were measured using an ELISA kit (Cat. No. EH0216 and Cat. No. EH0355, Fine Test, Wuhan, China), as previously described [9]. All the ELISA determinations were carried out employing a microplate reader spectrophotometer (InfiniteM200, Tecan, Austria).

Statistical analysis

Due to the small sample size, dCohen with 95%CI was utilized for calculating the size effect, and the corresponding value for each parameter was included. Furthermore, the coefficient of variation pre- and post-SF was computed. Percentage changes, defined as [(post – pre suborbital SF)/(pre suborbital SF) × 100], were used for the analysis to evaluate the effects of suborbital SF on the assessed biomarkers. Data are presented as mean ± SD. All measurements were performed in duplicate, and the inter-assay coefficient of variation was within the range indicated by the manufacturer.

References

1. European Space Agency (ESA) Astronaut Applicant Medical Examinations List. https://esamultimedia.esa.int/docs/eac/ESA_Astronaut_Applicant_Medical_Examinations_List.pdf.
2. Cannavo A, Jun S, Rengo G, Marzano F, Agrimi J, Liccardo D, et al. β3AR-dependent brain-derived neurotrophic factor (BDNF) generation limits chronic postischemic heart failure. Circ Res. 2023;132(7):867-81.
3. Bosco G, Giacon TA, Paolocci N, Vezzoli A, Noce CD, Paganini M, et al. Dopamine/BDNF loss underscores narcosis cognitive impairment in divers: a proof of concept in a dry condition. Eur J Appl Physiol. 2023;123(1):143-58.
4. Giacon TA, Bosco G, Vezzoli A, Dellanoce C, Cialoni D, Paganini M, et al. Oxidative stress and motion sickness in one crew during competitive offshore sailing. Sci Rep. 2022;12(1):1142.
5. Mrakic-Sposta S, Montorsi M, Porcelli S, Marzorati M, Healey B, Dellanoce C, et al. Effects of prolonged exposure to hypobaric hypoxia on oxidative stress: overwintering in antarctic concordia station. Oxid Med Cell Longev. 2022;2022:4430032.
6. Mrakic-Sposta S, Gussoni M, Marzorati M, Porcelli S, Bosco G, Balestra C, et al. The "ON-OFF" switching response of reactive oxygen species in acute normobaric hypoxia: preliminary outcome. Int J Mol Sci. 2023;24(4):4012.
7. Vezzoli A, Mrakic-Sposta S, Dellanoce C, Montorsi M, Vietti D, Ferrero ME. Chelation therapy associated with antioxidant supplementation can decrease oxidative stress and inflammation in multiple sclerosis: preliminary results. Antioxidants (Basel), 2023;12(7):1338.
8. Dorn LD, Lucke JF, Loucks TL, Berga SA. Salivary cortisol reflects serum cortisol: analysis of circadian profiles. Ann Clin Biochem. 2007;44(Pt 3):281-4.
9. Santangelo C, Verratti V, Mrakic-Sposta S, Ciampini F, Bonan S, Pignatelli P, et al. Nutritional physiology and body composition changes during a rapid ascent to high altitude. Appl Physiol Nutr Metab. 2024;49(6):723-37.

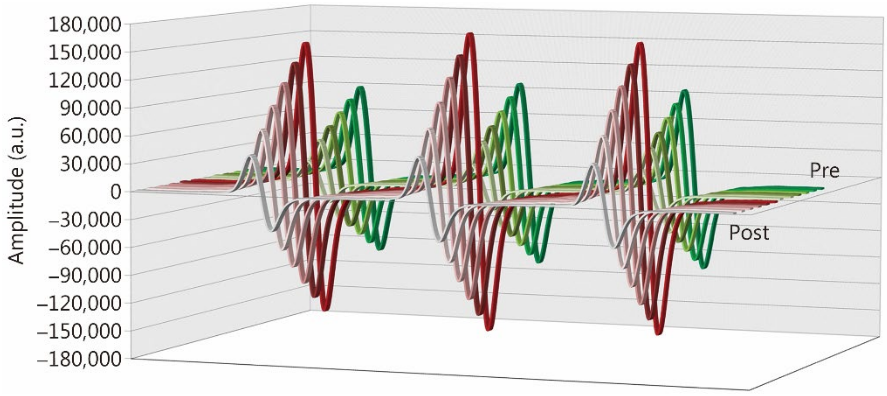

Fig. S1 Stacked plots of the reactive oxygen species (ROS) by electron paramagnetic resonance (EPR) spectra recorded at 37 °C, the signal comes from the reaction of 1-hydroxy-3-carboxymethyl-2,2,5,5-tetramethyl-pyrrolidine spin probe (CMH, EPR silent) to 3-carboxymethyl-2,2,5,5-tetramethyl-pyrrolidinyloxy radical (CM, EPR active). When the signal is sequentially acquired, the ROS production rate can be calculated. Using a stable radical compound as a reference like 3-carboxy-2,2,5,5-tetramethyl-1-pyrrolidinyloxy (CP) the absolute concentration levels are obtained
